# Supplementary material for: Simple physical mixing of zeolite prevents sulfur deactivation of vanadia catalysts for NOx removal
Source: Nat Commun. 2021 Feb 10;12:901. doi: 10.1038/s41467-021-21228-x (PMC7876025; doi:10.1038/s41467-021-21228-x)
Supplement: Supplementary file 1 — Supplementary Information [file 41467_2021_21228_MOESM1_ESM.pdf]

Supplementary Information for

Simple physical mixing of zeolite prevents sulfur deactivation of vanadia catalysts for NO_x removal

Inhak Song[1]†, Hwangho Lee[1]†, Se Won Jeon[1], Ismail A. M. Ibrahim[2,3], Joonwoo Kim[4], Youngchul Byun[4], Dong Jun Koh[4], Jeong Woo Han[2], Do Heui Kim[1]*

[1]School of Chemical and Biological Engineering, Institute of Chemical Processes, Seoul National University, Seoul 08826, Republic of Korea.

[2]Department of Chemical Engineering, Pohang University of Science and Technology (POSTECH) 77 Cheongam-ro, Nam-gu, Pohang, Gyeongbuk 37673, Republic of Korea.

[3]Department of Chemistry, Faculty of Science, Helwan University, Ain-Helwan, 11795 Cairo, Egypt.

[4]Research Institute of Industrial Science and Technology (RIST) 187-12, Geumho-ro, Gwangyang-si, Jeollanam-do, Republic of Korea.

*Corresponding author.
Phone: +82-2-880-1633
E-mail: dohkim@snu.ac.kr
†These authors equally contributed.

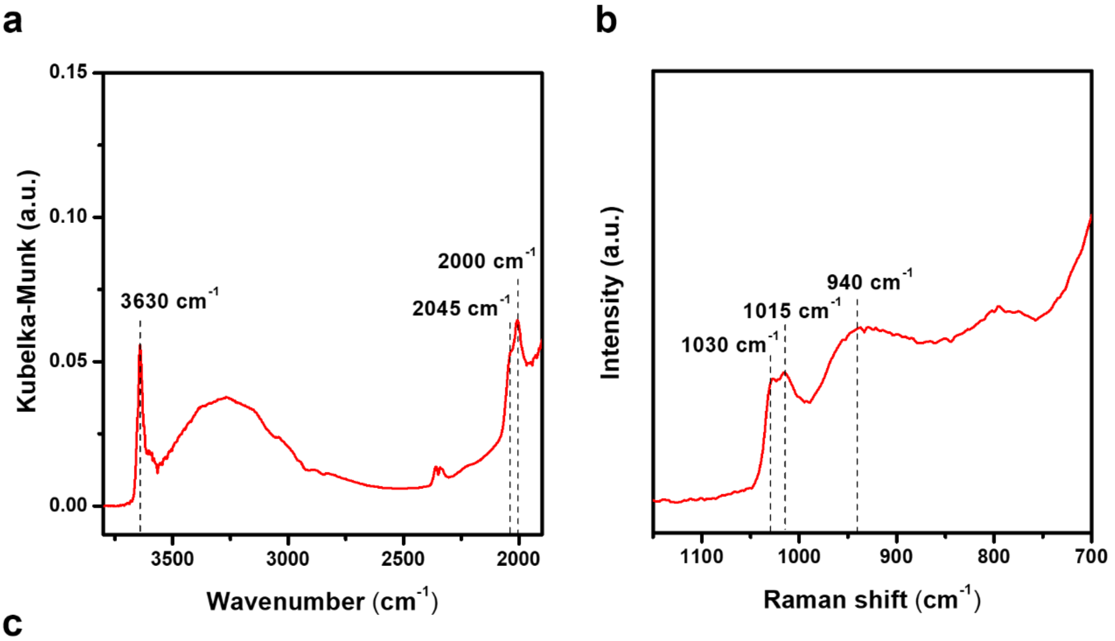

c

VWTi	V_2O_5	WO_3
**Content (wt %)**	4.91	9.71

Supplementary Fig. 1.

Characterization results of VWTi catalyst.

a, DRIFT spectrum of VWTi at ambient condition. Peak at 3630 cm^{-1} was hydroxyl group from V-OH and Ti-OH[1, 2]. Peaks at 2045 and 2000 cm^{-1} were assigned to vibration of M=O of vanadium oxide and tungsten oxide, respectively[2, 3]. **b,** Raman spectrum of VWTi at ambient condition. Peak at 1030 cm^{-1} and 1015 cm^{-1} were M=O vibration of vanadium oxide and tungsten oxide, respectively. Peak at 940 cm^{-1} was assigned to V-O-V vibration of vanadium oxide[3]. The vibration peak of V-O-V stands for presence of polymeric vanadia known for having higher SCR reactivity than monomeric vanadia. **c,** Content of vanadium and tungsten from ICP-AES of VWTi.

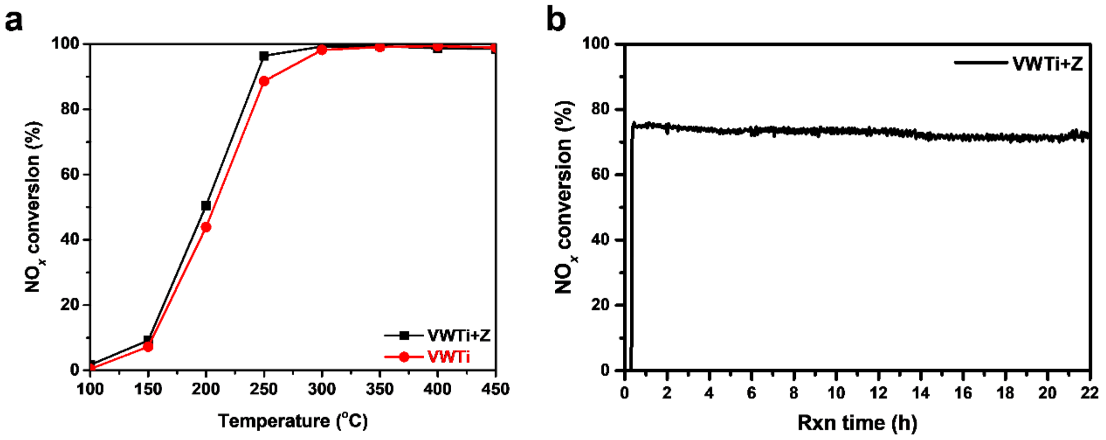

Supplementary Fig. 2.

Reaction test of VWTi and VWTi+Z.

a, Reactivity of VWTi and VWTi+Z at 100-450 °C under NH_3-SCR condition (500 ppm NO, 600 ppm NH_3, 10% O_2, 5% CO_2, 10% H_2O and balance N_2, GHSV=150,000 mL/h g catalyst of VWTi). VWTi and VWTi+Z showed almost same NH_3-SCR reactivity. It shows that physically mixed Y zeolite (Si/Al_2=12) has minor effect on NH_3-SCR reactivity. **b,** Reaction stability of VWTi+Z at 220 °C for 22 h under NH_3-SCR condition. There was no degradation of activity under NH_3-SCR condition without SO_2.

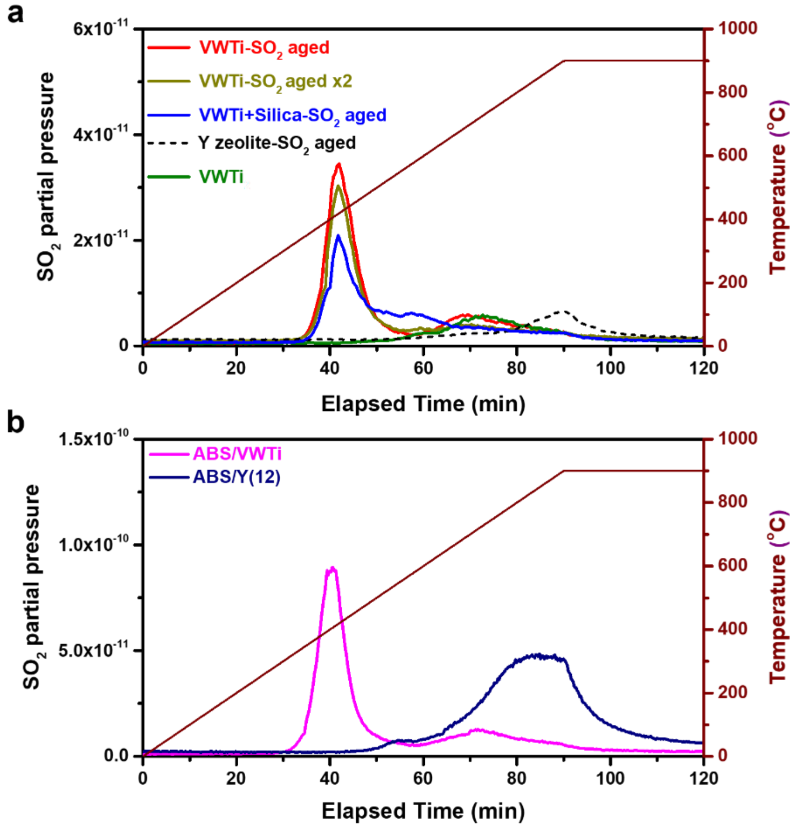

Supplementary Fig. 3.

Mass spectrometry results of SO_2 aged and ABS pre-impregnated samples during thermal decompositions.

TPD experiments were performed under N_2 flow of 100 mL/min with samples loading of 0.02 g. Temperature was set from ambient temperature to 900 °C at ramp rate of 10 °C/min. **a,** SO_2 profiles from TPD-Mass of sulfur aged VWTi, Y zeolite and physical mixed catalysts. A peak at 415 °C indicates SO_2 from a decomposition of ABS, and peak at ~700 °C is from a decomposition from stable metal sulfate[4, 5]. In the TPD-Mass of SO_2 aged VWTi (red), ABS deposition on the VWTi was observed, which was same result to previous studies. Second-aged VWTi (dark yellow) showed same SO_2 profiles to VWTi-SO_2 aged (red). It means that VWTi was completely regenerated by our regeneration condition at 350 °C. SO_2 profile of aged VWTi+Silica (blue) demonstrates that physically mixed silica could hardly protect the VWTi from blocking by ABS. SO_2 aged Y zeolite (black dash) hardly adsorbed SO_2 under SO_2 aging condition. SO_2 peak at 700 °C of the VWTi was assigned to a decomposition of titanium sulfate which existed on fresh VWTi (green). **b,** SO_2 profiles from TPD-Mass of ABS pre-impregnated VWTi and Y zeolite (Si/Al_2=12). Pre-impregnated ABS on VWTi (magenta) was decomposed at common decomposition temperature ~400 °C. However, ABS on Y zeolite (navy) showed totally different decomposition temperature which was assigned to decomposition of metal sulfate (700-800 °C). It can be inferred that remained sulfate might form metal sulfate, which was supposed to be aluminum sulfate species by an interaction of sulfate to zeolite Al sites.

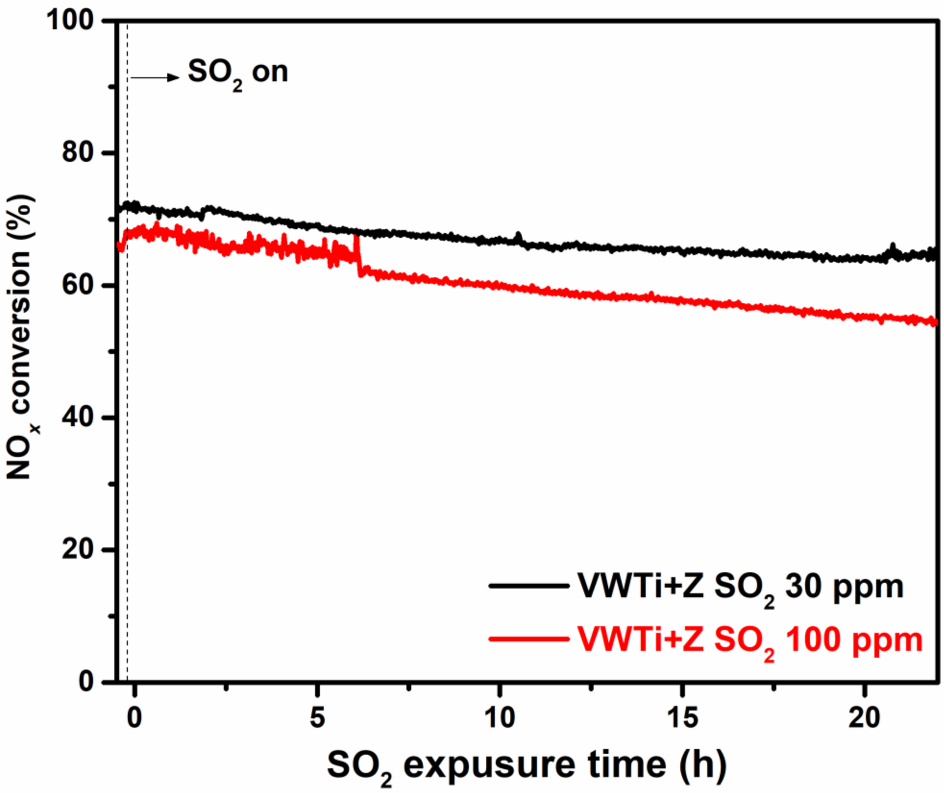

Supplementary Fig. 4.

Reaction test under SO_2 in higher concentration.

Reaction profiles of 22 h SO_2 aging over VWTi+Z under NH_3-SCR condition with di fferent SO_2 concentration at 220 °C (500 ppm NO, 600 ppm NH_3 10% O_2, 5% CO_2, 10% H_2O, 30 ppm or 100 ppm SO_2 and balanced with N_2, GHSV : 150,000 mL/h·g_{c} $_{at}$ based on VWTi weight).

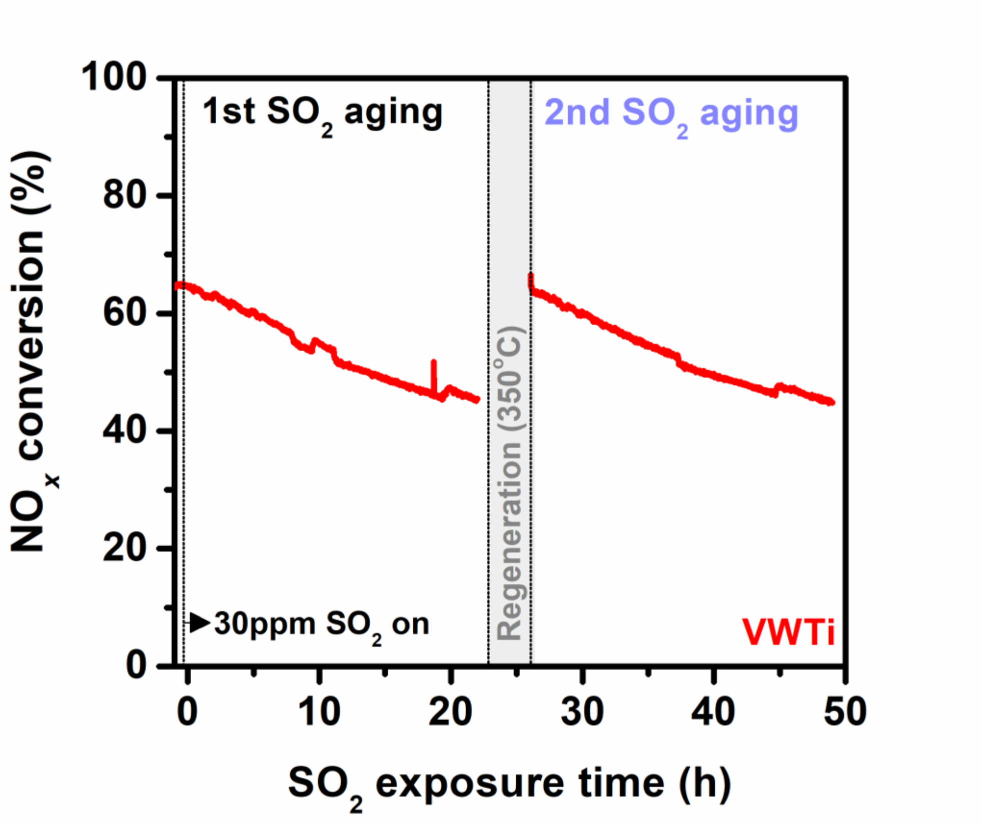

Supplementary Fig. 5.

Regeneration and reusability test of VWTi catalyst after SO_2 aging.
The catalyst was aged under 500 ppm NO, 600 ppm NH_3, 10% O_2, 5% CO_2, 10% H_2O, 30 ppm SO_2 and balance N_2. Regeneration condition was 10% O_2, 5% CO_2, 10% H_2O and balance N_2. VWTi catalyst was totally regenerated under the 350 °C thermal treatment.

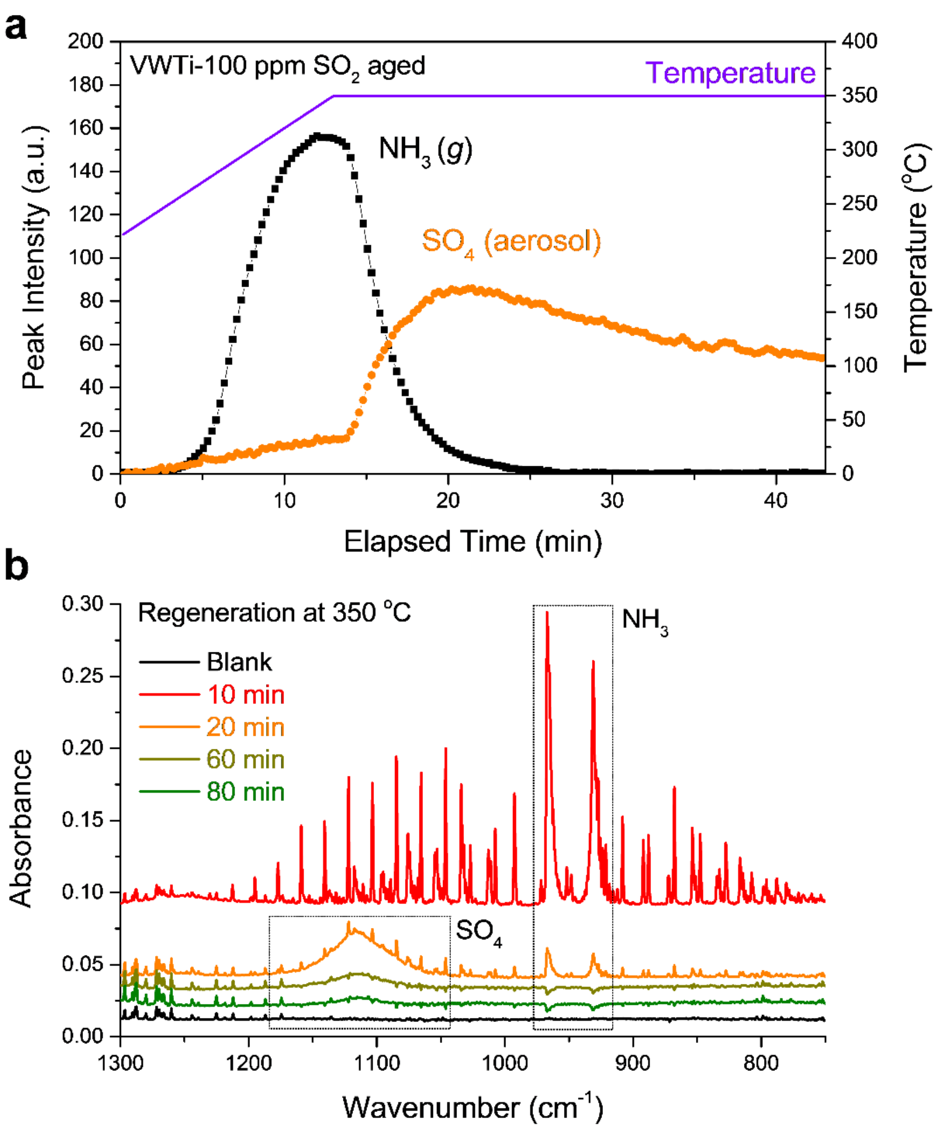

Supplementary Fig. 6.

Outlet gas profiles of VWTi catalyst during regeneration step.

a, Ammonia and sulfate profiles during regeneration step which were obtained by using FT-IR with a gas cell. Before regeneration, VWTi catalyst was aged under SCR reaction gas containing 100 ppm SO_2 at 220 °C for 22 h. Then, the reactor was heated from 220 to 350 °C with a ramp rate of 10 °C/min and held for 2 h under regeneration gas. Regeneration gas contained 10% O_2, 5% CO_2, 10% H_2O balanced with N_2. **b**, Selected FT-IR spectra during regeneration of catalyst.

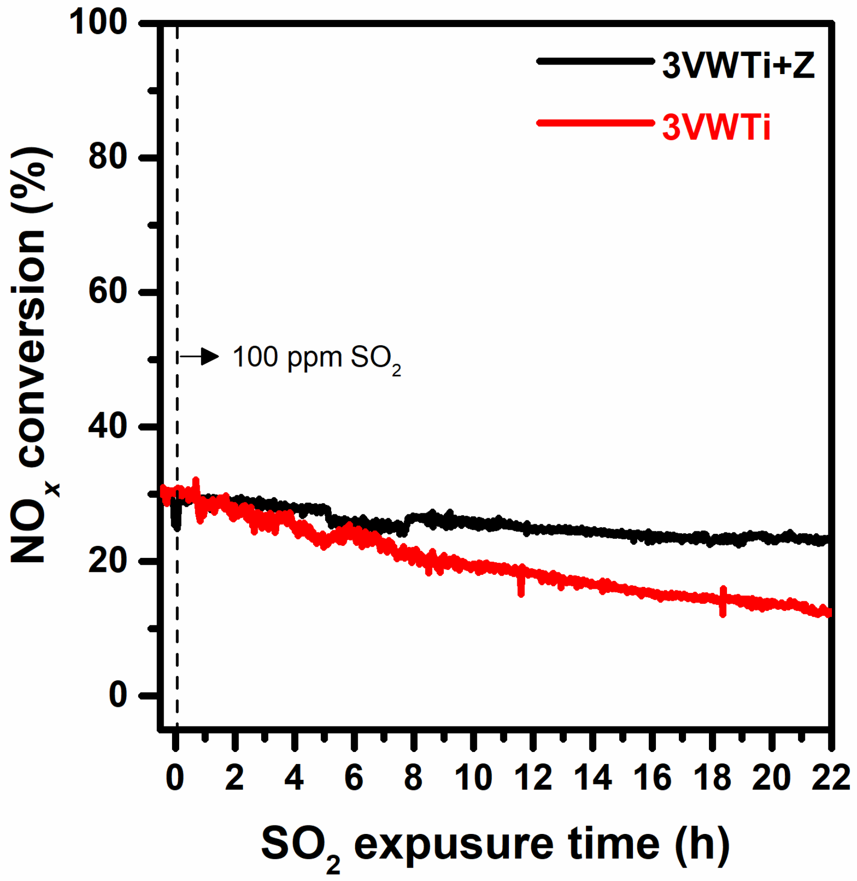

Supplementary Fig. 7.

Reaction tests under 100 ppm SO_2 for VWTi catalyst with 3 wt.% V_2O_5.
Reaction profiles of 22 h SO_2 aging over 3 wt.% V_2O_5/WO_3-TiO_2 catalyst (3VWTi) a nd its mixture with Y zeolite (3VWTi+Z) under NH_3-SCR condition at 220 °C (500 ppm NO, 600 ppm NH_3 10% O_2, 5% CO_2, 10% H_2O, 100 ppm SO_2 and balanced w ith N_2, GHSV : 150,000 mL/h·g_{cat} based on VWTi weight).

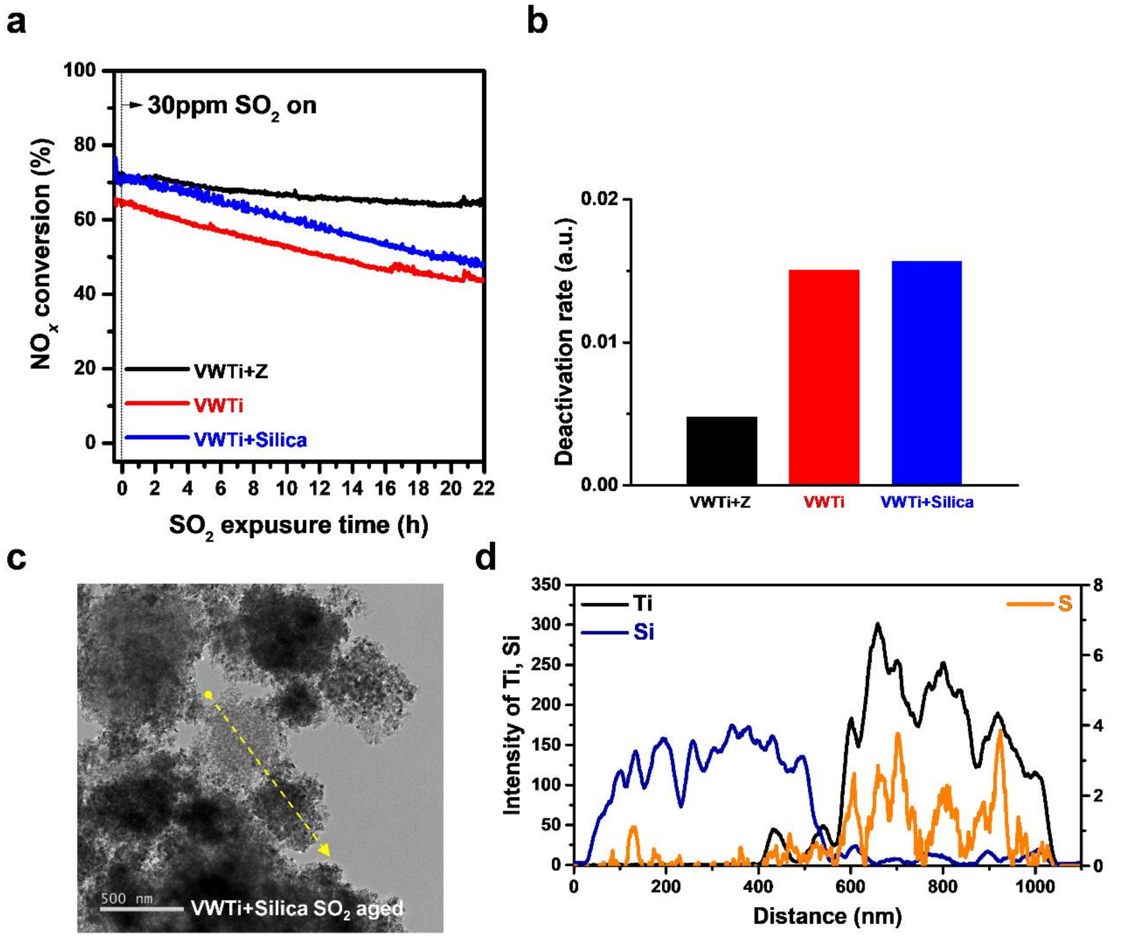

Supplementary Fig. 8.

Physical mixture of VWTi and Silica in a 2:1 mass ratio.

a, b, Comparison of sulfur resistance of VWTi, VWTi+Z and VWTi+Silica under the SO_2 aging condition. VWTi+Silica showed almost same deactivation rate to VWTi which was three times larger than VWTi+Z. It demonstrates that the physical mixed silica has little effect on enhancing sulfur resistance of VWTi in contrast to the physical mixed zeolite. **c, d,** TEM image and line-EDS of VWTi+Silica after SO_2 aging. In a sulfur distribution from line-EDS, most of sulfur was observed in VWTi region, which was same to VWTi. This result illustrates that the silica cannot absorb the sulfur in contrast to the zeolite, therefore, cannot enhance the sulfur resistance.

a

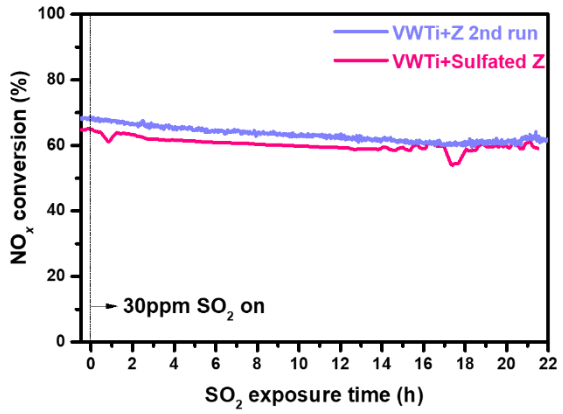

b

	Al (wt%)	S (wt%)	S/Al (molar ratio)
Sulfated Z	5.33	9.03	1.43

c d

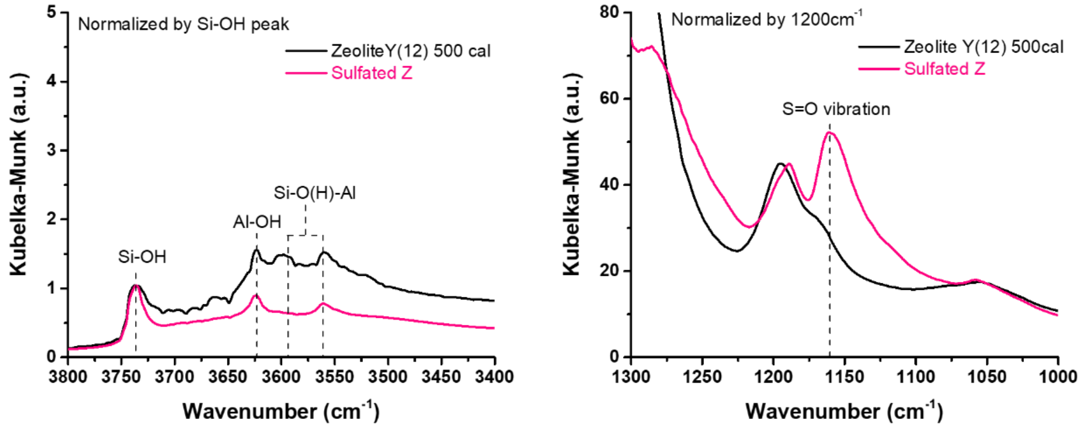

Supplementary Fig. 9.

Physical mixture of VWTi and sulfated Y zeolite (Sulfated Z) in a 2:1 mass ratio.
60 wt.% of ABS was impregnated on Y zeolite (Si/Al_2=12) and calcined at 500 °C for 4h to prepare Sulfated Z. **a,** Comparing sulfur resistance of VWTi+Sulfated Z to that of VWTi+Z. **b,** Deposited amount of sulfur on Sulfated Z. Although ABS is commonly decomposed under 350 °C, sulfur still remained in the zeolite due to its strong interaction to zeolite after calcined at 500 °C. This species might be aluminum sulfate like species with interaction to zeolite Al sites. Sulfur amount was 9.03 wt.%, which is 1.43 as S/Al ratio indicating that Al sites of the Y zeolite were nearly saturated to sulfur. **c, d,** DRIFT spectra of the Y zeolite and Sulfated Z at 250 °C after pretreatment at 400 °C under air condition using KBr background. Peaks at 3800-3400 cm^{-1} demonstrated vibrations of hydroxyl group (-OH). Peaks at 3730, 3624 cm^{-1} are assigned to terminal Si-OH, Al-OH, respectively. Peaks at 3590 and 3567 cm^{-1} are vibration of Si-OH-Al which originates from protons on framework ion exchange sites of zeolite[6]. In the Sulfated Z, hydroxyl group from alumina decreased compared to fresh Y zeolite. And S=O vibration peak at 1162 cm^{-1} was observed, which means that sulfate species was deposited on the Sulfated Z[7]. From this results, it can be inferred that the remained sulfate interacted to zeolite Al as a form of aluminum sulfate like species in the Sulfated Z.

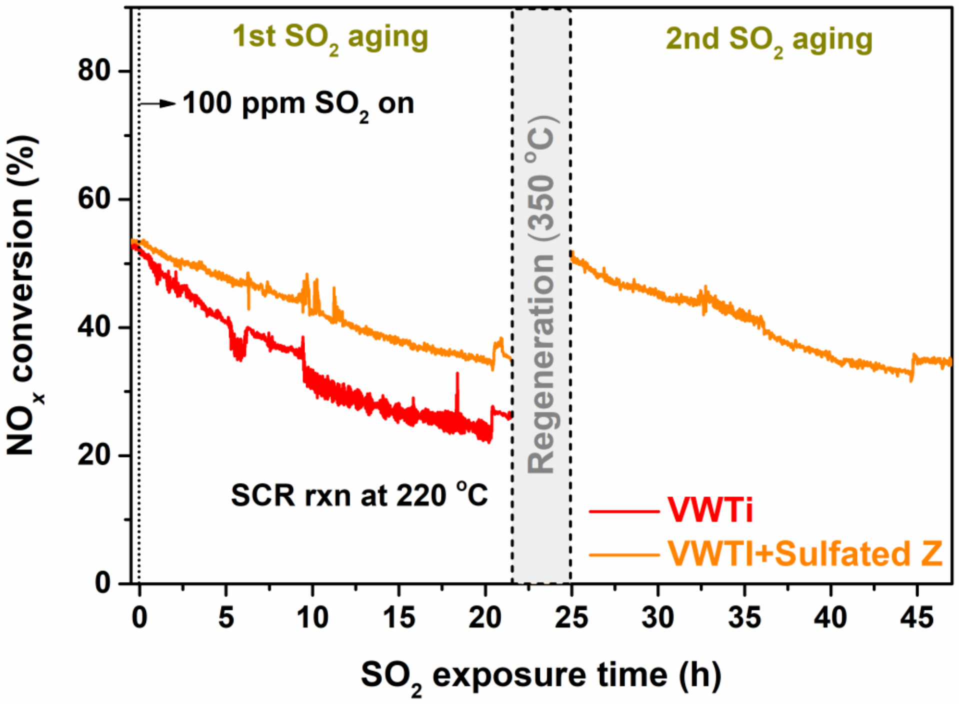

Supplementary Fig. 10.

Regeneration and reusability test of VWTi catalyst after SO_2 aging.

The catalyst was aged under 500 ppm NO, 600 ppm NH_3, 10% O_2, 5% CO_2, 10% H_2O, 30 ppm SO_2 and balance N_2. Regeneration condition was 10% O_2, 5% CO_2, 10% H_2O and balance N_2. VWTi catalyst was totally regenerated under the 350 °C thermal treatment.

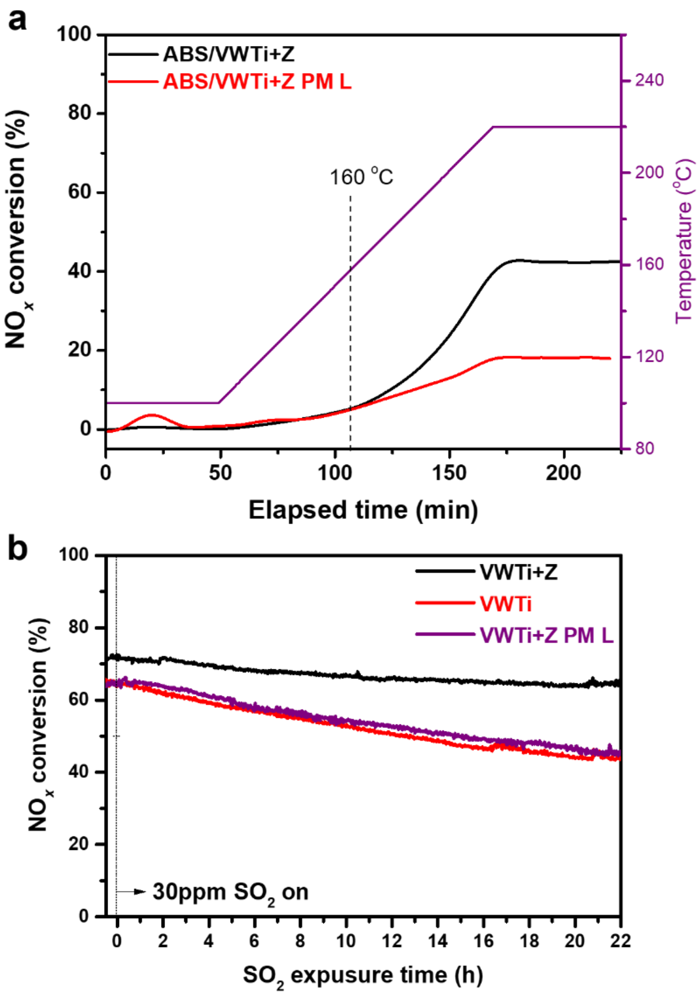

Supplementary Fig. 11.

Comparison of physically mixed VWTi and Y zeolite (VWTi+Z) to loose contact sample (VWTi+Z PM L).

a, NO_x conversion data of ABS/VWTi+Z and PM L were obtained from transient NH_3-SCR reaction at temperature from 100 to 220 °C with ramp rate 1 °C/min. ABS/VWTi+Z PM L had reactivity of 45 % NO_x conversion at 220 °C indicating deactivation by impregnated ABS. In contrast, ABS/VWTi+Z showed almost same activity to fresh VWTi (NO_x conversion 59% at 220 °C). It means that physical contact mitigated the deactivation of VWTi which was blocked by impregnated ABS. **b,** 22 h SO_2 aging profiles of VWTi+Z PM L under NH_3-SCR reaction condition with 30 ppm SO_2 at 220 °C. NH_3-SCR condition was under 500 ppm NO, 600 ppm NH_3, 10% O_2, 5% CO_2, 10% H_2O and balance N_2. GHSV: 150,000 mL/h·g_{cat} based on VWTi weight.

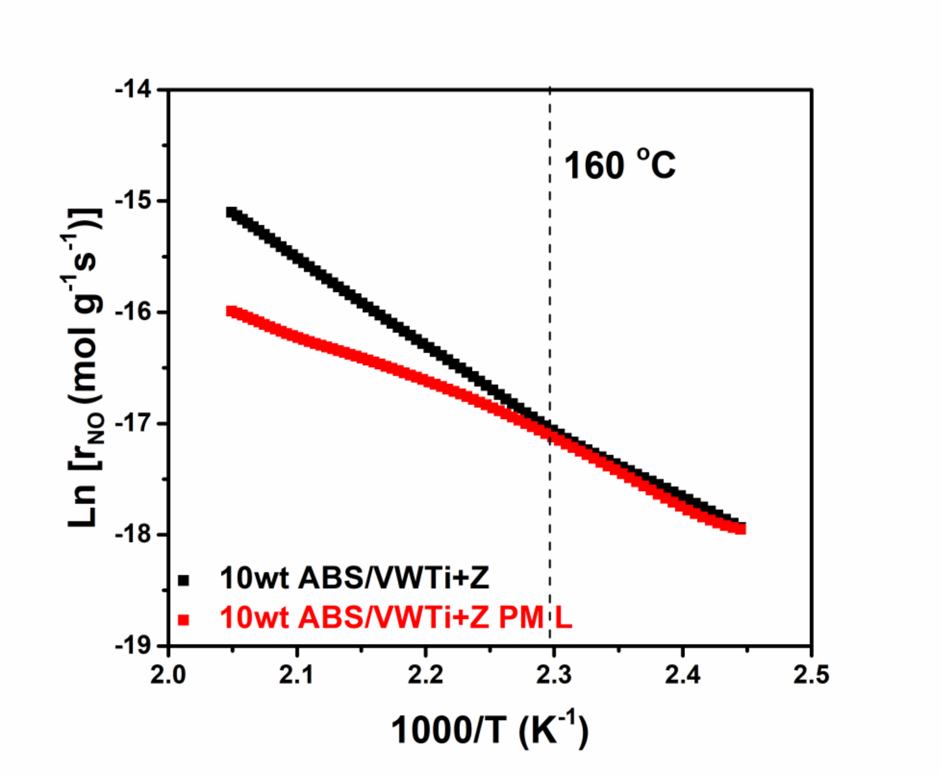

Supplementary Fig. 12.

Arrhenius plots of 10wt ABS/VWTi+Z (black dot) and 10wt ABS/VWTi+Z PM L (red dot) during transient NH_3-SCR reaction (temperature from 100 °C to 220 °C with ramp rate 1 °C/min, other reaction conditions were the same as steady-state NH_3-SCR experiment. The 10wt ABS/VWTi sample was prepared by wet impregnation method of 10 wt.% of ABS on the VWTi catalyst.

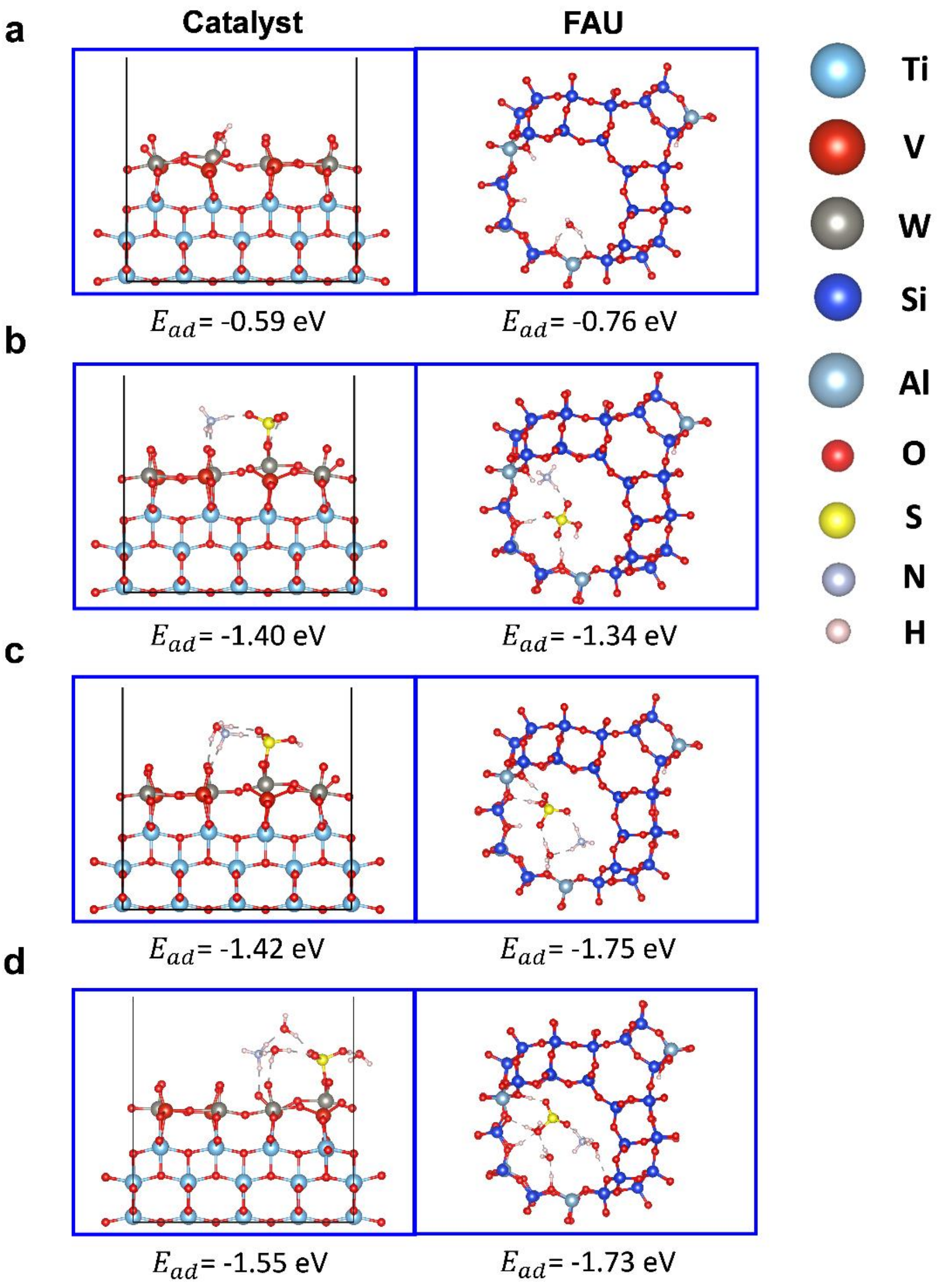

Supplementary Fig. 13.

Optimized configurations used in DFT calculation.

a, Water adsorption energy on the VWTi catalyst and H-Y zeolite was calculated. **b,** Optimized configurations of ABS molecule without water on the VWTi and H-Y zeolite. ABS molecule is strongly bounded to catalyst surface through HO_3S–O···W chemical bond on the VWTi. In the case of H-Y zeolite, ABS molecule is stabilized through the bonding with Brønsted acid sites. **c,** Optimized configurations for hydrated ABS with one water molecule. **d,** Optimized configurations for hydrated ABS with three water molecules.

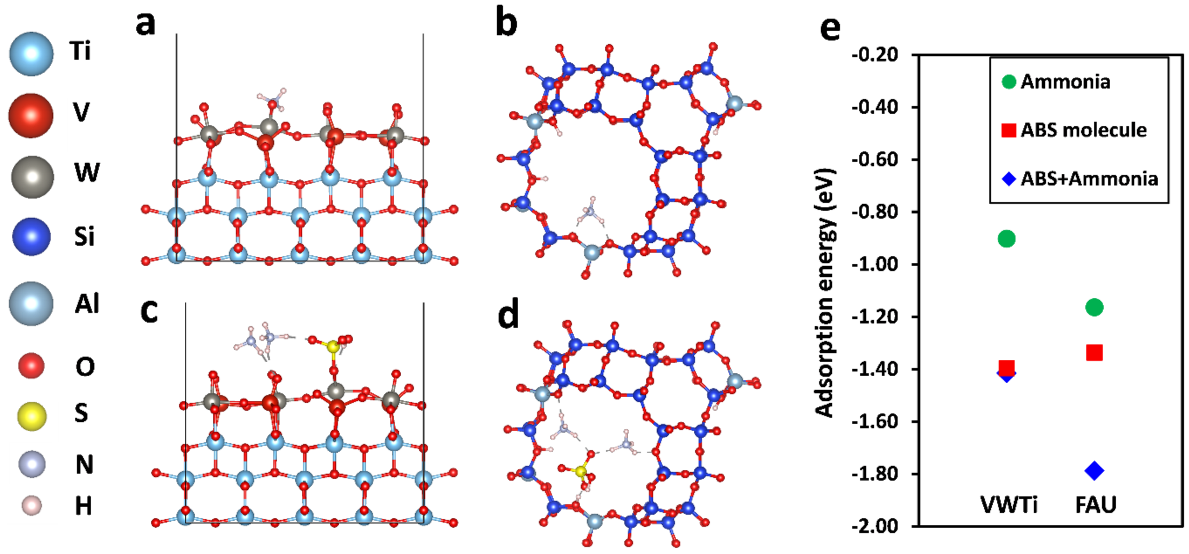

Supplementary Fig. 14.

Optimized configurations used in DFT calculation.
a-b, Optimized configurations of ammonia adsorbed on the VWTi catalyst and H-Y zeolite. **c-d**, Optimized configurations of ABS molecule with pre-adsorbed ammonia on the VWTi catalyst and H-Y zeolite. **e**, Comparison of the calculated adsorption energy.

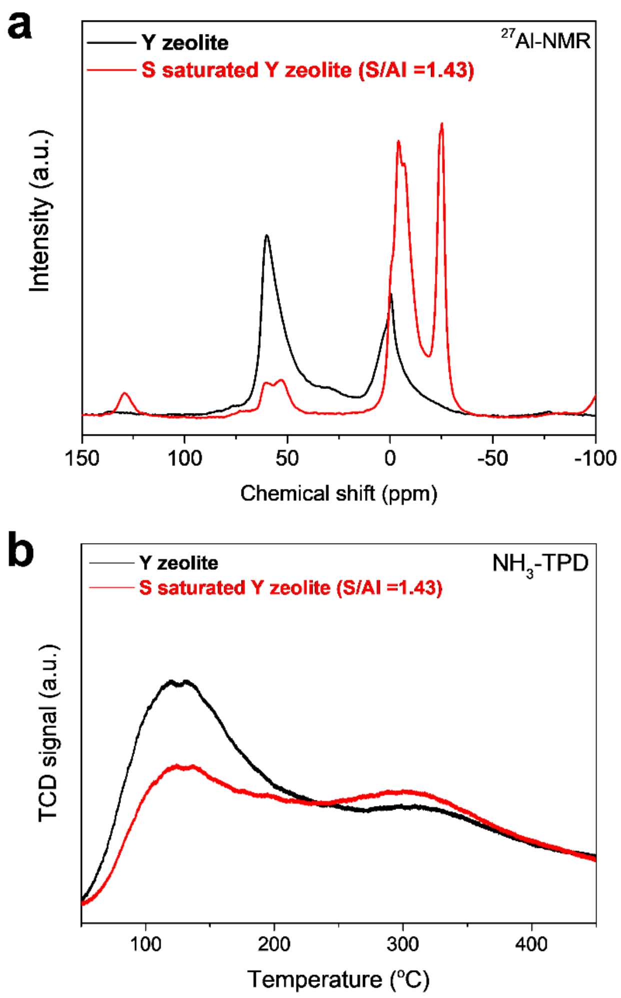

Supplementary Fig. 15.

Characteristics of Y zeolite and sulfur saturated Y zeolite (Sulfated Z).

a, ^{27}Al-NMR spectra of Y zeolite and sulfur saturated Y zeolite. **b**, NH_3-TPD profiles of Y zeolite and sulfur saturated Y zeolite. NH_3 adsorbed at 50 °C for NH_3 saturation and purged under He. Then, the samples were ramped to 500 °C with the rate of 5 °C/min. The resonance peaks at 55 ppm and 0 ppm are assigned to framework Al and extra-framework Al, respectively[8, 9]. The peak at 55 ppm decreased dramatically in sulfur saturated Y zeolite, which indicates the strong interaction between sulfate and framework Al. To investigate acidic properties of the Y zeolite and sulfur saturated Y, the NH_3-TPD experiment was performed. It is well known that the Y zeolite shows two NH_3 desorption peaks at low and high temperature (120 and 300 °C, respectively) assigned to weakly bound NH_3 on Lewis acid sites and NH_3 on Brønsted acid sites, respectively. Although the sulfate was bound to framework Al, there was little change in the number of Brønsted acid sites in sulfur saturated Y zeolite compared to the pristine Y zeolite, probably because the sulfated sites can also act as the Brønsted acid sites. As a result, VWTi+Z catalyst demonstrates the almost same performance during a series of aging and regeneration cycle.

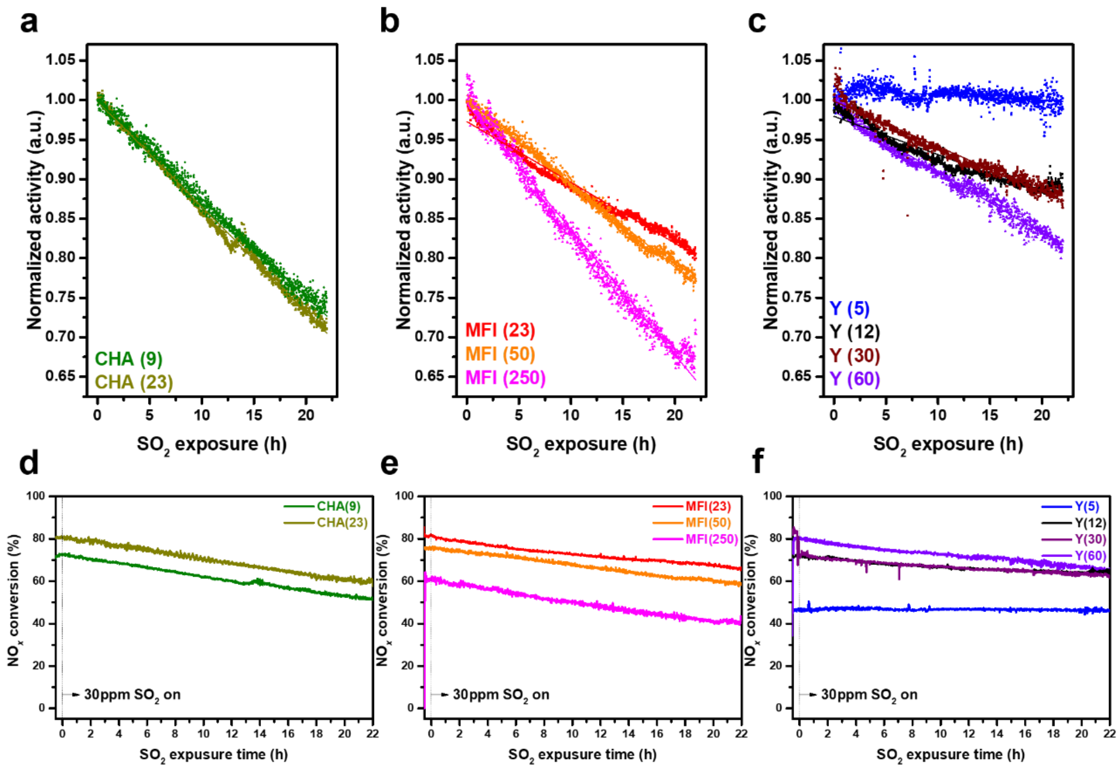

Supplementary Fig. 16.

SO_2 aging profiles of physically mixed VWTi and various zeolite.

a-c, Deactivation profiles normalized by initial activity during 22 h SO_2 aging. **d-f,** NO_x conversion profiles of physical mixture during 22 h SO_2 aging. SO_2 aging condition was under 500 ppm NO, 600 ppm NH_3, 10% O_2, 5% CO_2, 10% H_2O, 30 ppm SO_2 and balance N_2. GHSV: 150,000 mL/h ·g_{cat} based on VWTi weight. SSZ-13, ZSM-5 and Y zeolite were used for physical mixing with VWTi, whose structures were CHA, MFI and FAU, respectively.

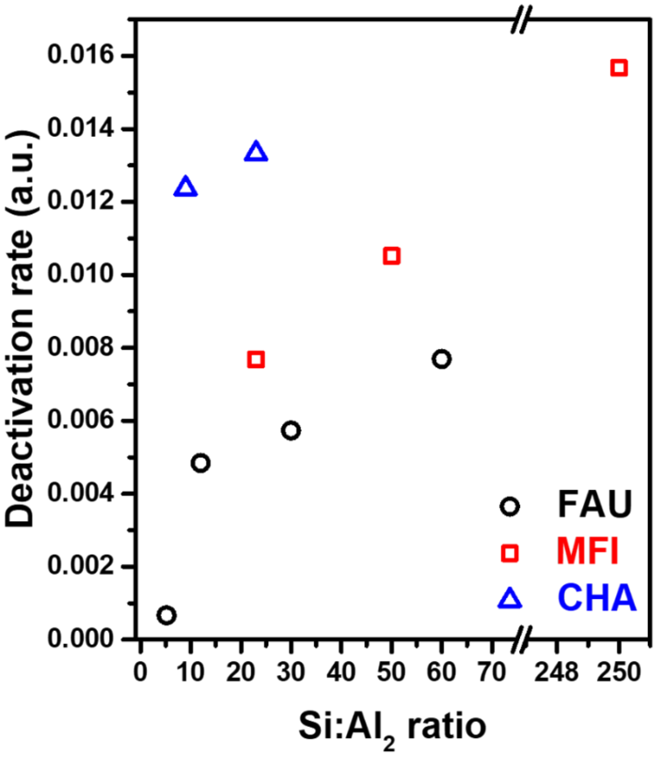

Supplementary Fig. 17.

Correlation of deactivation rate to Si/Al_2 ratio and zeolite structure.

Deactivation rates were obtained from Supplementary Table 4, and the Si/Al_2 ratio values written on commercial zeolites were used. Deactivation rate decreased as decreasing Si/Al_2 ratio of mixed zeolite. Comparing zeolites which have similar Si/Al_2 ratio (FAU: 30, MFI: 23, CHA: 23), deactivation rate decrease by CHA, MFI, FAU.

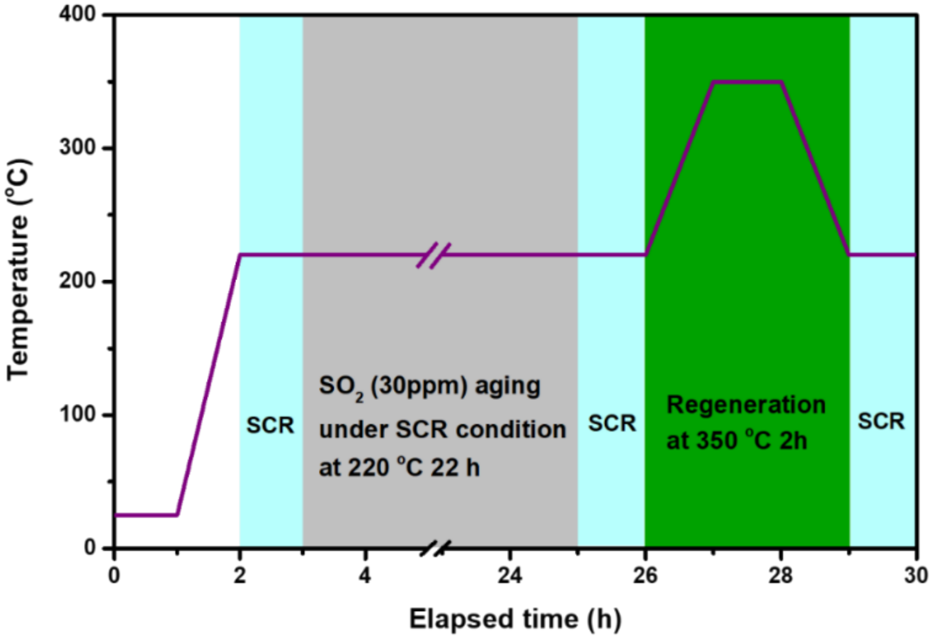

Supplementary Fig. 18.
The protocol for simulating sulfur aging and subsequent regeneration experiments.

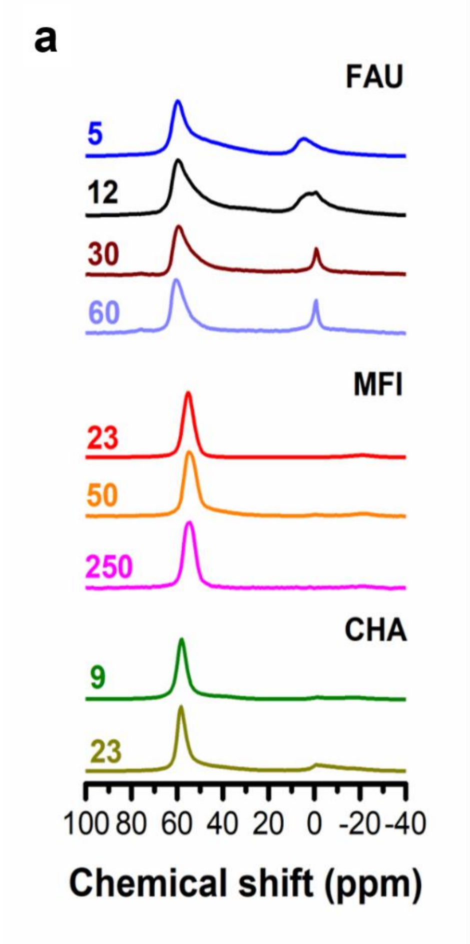

b

Structure	Si/Al_2	$Al_{framework}/Al_{total}$
FAU	5	0.751
	12	0.675
	30	0.744
	60	0.735
MFI	23	1
	50	1
	250	1
CHA	9	0.963
	23	0.819

Supplementary Fig. 19.

Solid-state ^{27}Al-NMR spectra and quantification data of zeolites used for physical mixing.

a, Solid-state ^{27}Al-NMR spectra for various zeolites mixed with VWTi catalyst in this work. **b,** Amount of framework Al was calculated from integrated area of 4-coordinated Al peak at around 60 ppm, and extraframework Al from integrated area of peak at 0 ppm which indicates 6-coordinated Al. $Al_{framework}/Al_{total}$ was proposed as $Al_{framework}/(Al_{framework}+Al_{extraframework})$.

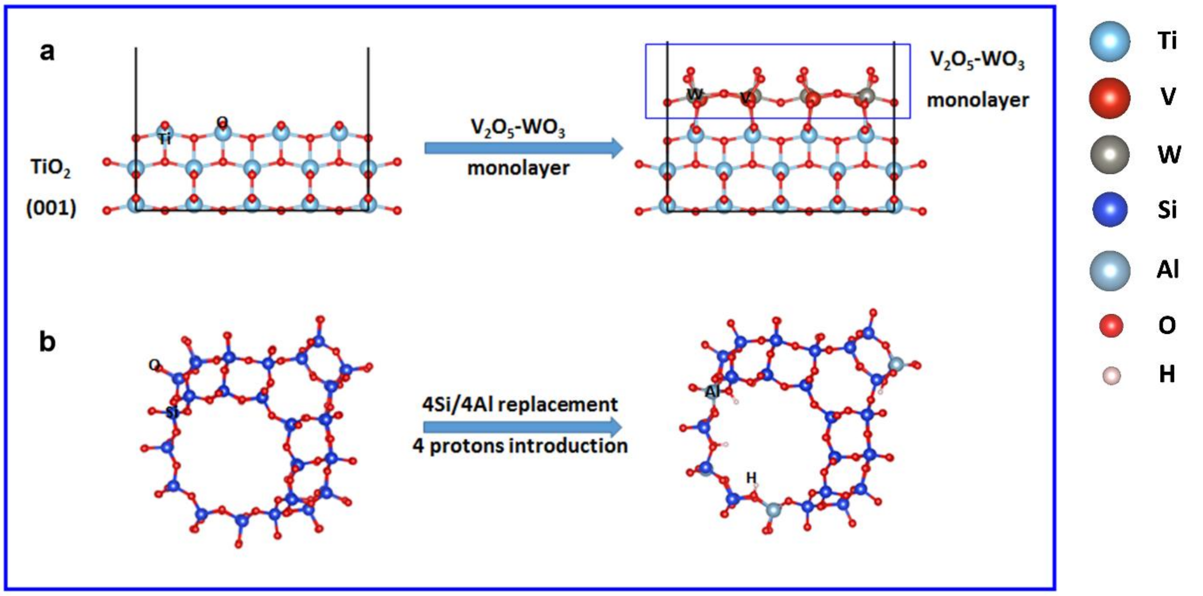

Supplementary Fig. 20.

a, Schematic representation of loading of vanadia and tungsten oxide monolayer on TiO_2 support. **b,** Schematic representation of constructing of FAU zeolite model with Si/Al ratio of 11.

Supplementary Table 1.

Deposition amount of sulfur on SO_2 aged samples. Sulfur amount was quantified by elemental analysis and converted to sulfur amount per g catalyst of VWTi.

Samples	Deposited amount of S (wt. % /VWTi)
VWTi SO_2 aged	0.3801
VWTi+Z SO_2 aged	0.5652
VWTi+Z SO_2 aged x2	0.7715
VWTi+Z SO_2 aged x3	1.3286

Supplementary Table 2.

Activation energy (E_a) and pre-exponential factor obtained from the Arrhenius plots of ABS/VWTi+Z and ABS/VWTi+Z PM L (2wt.% ABS) during the transient SCR reaction

		< 160 °C	> 180 °C
ABS/VWTi+Z	E_a (kJ/mol)	70.4	61.1
	A	2.1 x 10^7	1.8 x 10^6
ABS/VWTi+Z PM L	E_a (kJ/mol)	70.4	58.3
	A	2.0 x 10^7	6.7x10^5

Below the melting point of ABS (160 °C), the ABS/VWTi+Z and ABS/VWTi+Z PM L show same activation energy (E_a = 70.4 kJ/mol) and pre-exponential factor values (A = 2.1 x10^7 and 2.0 x 10^7) because VWTi catalyst cannot be deactivated when impregnated ABS is present as solid. Above 180 °C, the both catalysts still show almost same E_a value of 61.1 and 58.3 kJ/mol. However, the pre-exponential factor (A) decreased from 1.8 x 10^6 to 6.7 x 10^5 in the ABS/VWTi+Z PM L, which clearly indicates that the impregnated ABS physically deactivated the VWTi catalyst.

Supplementary Table 3.

Linear fitting results of reaction mechanism investigation over various zeolites in Fig. 4.

Zeolite (structure)	Y zeolite (FAU)	ZSM-5 (MFI)	SSZ-13 (CHA)
Slope ($-k_m$)	0.1395 ± 0.0168	0.0946 ± 0.0093	0.0130 ± 0.0049
Intercept	-4.3187	-4.1342	-4.1837
R^2	0.9446	0.9715	0.7533

Supplementary Table 4.

Deactivation rates of VWTi+zeolite physical mixture system. SSZ-13 (CHA), ZSM-5 (MFI) and Y zeolite (FAU) were used with a few kinds of Si/Al_2 ratios. Deactivation rates were derived from slope of linear fitting results of normalized deactivation profiles in Supplementary Fig. 15.

Zeolite (structure)	SSZ-13 (CHA)		ZSM-5 (MFI)			Y zeolite (FAU)			
Si/Al_2 ratio	9	23	23	50	250	5	12	30	60
Deactivation rate (h^{-1})	0.01236	0.01332	0.00768	0.01052	0.01567	6.663 x 10^{-4}	0.00484	0.00573	0.00769

Supplementary References

1. Topsøe, N.-Y. Mechanism of the selective catalytic reduction of nitric oxide by ammonia elucidated by in situ on-line Fourier transform infrared spectroscopy. *Science* **265**, 1217-1219 (1994).
2. Ramis, G., Bregani, F. & Forzatti, P. Fourier transform-infrared study of the adsorption and coadsorption of nitric oxide, nitrogen dioxide and ammonia on vanadia-titania and mechanism of selective catalytic reduction. *Appl. Catal.* **64**, 259-278 (1990).
3. Zhu, M., Lai, J.K., Tumuluri, U., Wu, Z. & Wachs, I.E. Nature of Active Sites and Surface Intermediates during SCR of NO with NH_3 by Supported V_2O_5--WO_3/TiO_2 Catalysts. *J. Am. Chem. Soc.* **139**, 15624-15627 (2017).
4. Chen, Y., Li, C., Chen, J. & Tang, X. Self-Prevention of Well-Defined-Facet Fe_2O_3/MoO_3 against Deposition of Ammonium Bisulfate in Low-temperature NH_3-SCR. *Environ. Sci. Technol.* (2018).
5. Guo, K. et al. Pore Size Expansion Accelerates Ammonium Bisulfate Decomposition for Improved Sulfur Resistance in Low-Temperature NH_3-SCR. *ACS Appl. Mater. Inter.* **11**, 4900-4907 (2019).
6. Lónyi, F., Valyon, J., Pál-Borbély, G. A DRIFT spectroscopic study of the N_2 adsorption and acidity of H-faujasites. *Micropor. Mesopor. Mat.* **66**, 273-282 (2003).
7. Davydov, A.A., Kantcheva, M. & Chepotko, M.L. FTIR spectroscopic study on nickel (II)-exchanged sulfated alumina: nature of the active sites in the catalytic oligomerization of ethene. *Catal. Lett.* **83**, 97-108 (2002).
8. Schmieg, S.J. et al. Thermal durability of Cu-CHA NH_3-SCR catalysts for diesel NO_x reduction. *Catal. Today* **184**, 252-261 (2012).
9. Coster, D., Blumenfeld, A. & Fripiat, J. Lewis acid sites and surface aluminum in aluminas and zeolites: a high-resolution NMR study. *J. Phys. Chem.* **98**, 6201-6211 (1994).
